# Impact on sales of adding a smaller serving size of beer and cider in licensed premises: an A-B-A reversal design

**DOI:** 10.1186/s12889-023-16163-z

**Published:** 2023-06-26

**Authors:** Eleni Mantzari, Minna Ventsel, Emily Pechey, Ilse Lee, Mark Pilling, Gareth J. Hollands, Theresa M. Marteau

**Affiliations:** 1 Behaviour and Health Research Unit, University of Cambridge, Cambridge, UK; 2 EPPI Centre, UCL Social Research Institute, University College London, London, UK

**Keywords:** Alcohol, Beer, Cider, Consumption, Serving size, Portion size, Pub, Bar, Restaurant, Field study

## Abstract

**Background:**

Smaller serving sizes of alcoholic drinks could reduce alcohol consumption across populations thereby lowering the risk of many diseases. The effect of modifying the available range of serving sizes of beer and cider in a real-world setting has yet to be studied. The current study assessed the impact on beer and cider sales of adding a serving size of draught beer and cider (2/3 pint) that was between the current smallest (1/2 pint) and largest (1 pint) standard serving sizes.

**Methods:**

Twenty-two licensed premises in England consented to taking part in the study. The study used an ABA reversal design, set over three 4-weekly periods, with A representing the non-intervention periods, during which standard serving sizes were served and B the intervention period when a 2/3 pint serving size of draught beer and cider was added to the existing range, along with smaller 1/2 pint and larger 1 pint serving sizes. The primary outcome was the daily volume of beer and cider sold, extracted from sales data.

**Results:**

Fourteen premises started the study, of which thirteen completed it. Twelve of those did so *per protocol* and were included in the primary analysis. After adjusting for pre-specified covariates, the intervention did not have a significant effect on the volume of beer and cider sold per day (3.14 ml; 95%CIs -2.29 to 8.58; *p* = 0.257).

**Conclusions:**

In licensed premises, there was no evidence that adding a smaller serving size for draught beer and cider (2/3 pint) when the smallest (1/2 pint) and largest (1 pint) sizes were still available, affected the volume of beer and cider sold. Studies are warranted to assess the impact of removing the largest serving size.

**Trial registration:**

ISRCTN: https://doi.org/10.1186/ISRCTN33169631 (08/09/2021), OSF: https://osf.io/xkgdb/ (08/09/2021).

**Supplementary Information:**

The online version contains supplementary material available at 10.1186/s12889-023-16163-z.

## Background

Excess alcohol consumption contributes to premature mortality and preventable morbidity [1], causing approximately three million global deaths per year and accounting for 5.1% of the global burden of disease [2]. Reducing alcohol consumption at the population level has been declared a global public health priority [3]. This is reflected in WHO Europe’s recent decision to commit all member states to a comprehensive plan for accelerating action on reducing alcohol consumption across the continent [4, 5]. Although recent years have seen a shift in the setting in which most alcohol is consumed, with more being consumed in homes rather than bars, pubs and restaurants [6], a large proportion of alcohol is still being consumed in licensed premises. For example, in 2019, almost a third of alcohol sold in Great Britain was bought and drunk in pubs, clubs, bars and restaurants [7], making licensed premises an important target for intervention.

Alcohol consumption in populations is influenced by a variety of factors, both at the individual, and environmental or contextual level [2, 8]. Individual factors include gender [9], family circumstances [10–12], social support [13] and socio-economic status [14]. Environmental factors include advertising, marketing, and product labelling, as well as opportunities for alcohol purchasing and consumption [15, 16] . For example, studies have shown that exposure to alcohol advertising can increase alcohol consumption [17–19], while restricting advertising can have a small impact on reducing consumption [20, 21]. Additional labelling of alcohol products, including health warning and calorie labels, may also have the potential to reduce alcohol use [22–24]. Furthermore, the physical availability of alcohol can influence consumption: the more readily alcohol is available, the more likely it is to be consumed [25]. In support of this, studies have shown that individuals living in neighbourhoods with more licensed premises, such as bars, tend to drink more [26, 27]. Decreasing the opportunity to buy alcohol by reducing its availability or its affordability can also reduce its consumption. For example, within retail settings, decreasing the proportion of alcoholic drinks for sale and increasing the proportion of non-alcoholic drinks—from 25% to 50% or 75%—can reduce the amount of alcohol selected and purchased [28]. Finally, there is a lot of evidence showing that increasing the affordability of alcohol by making it cheaper, increases its consumption [29, 30]*.* Conversely, decreasing its affordability decreases its consumption [31].

Interventions that involve changing the size of portions and containers of products that can harm health, including alcohol, also show promise. This follows from the well documented “portion size effect” for food, i.e. that people consume less when presented with smaller portions, packages, or tableware [32–34]. Until recently this effect had been neglected as a focus of study in relation to alcoholic drinks and the size of servings and containers in which these are served. There is now evidence showing that larger wine glasses increase the volume of wine sold, and therefore consumed, in restaurants [35]. Smaller wine glasses might also reduce the amount of wine drunk in homes, although the evidence for this is very limited [36]. Drinking wine at home from smaller 50cl bottles, compared with standard 75cl bottles, may also reduce consumption [37], but the impact of 37.5cl bottles is less certain [36].

Interventions that target the sizes of servings for reducing alcohol consumption can be classified broadly into three groups [38]:

i. removing the largest serving size from existing options;
ii. reducing the smallest serving size (either by adding a new smaller size or reducing the existing smallest size);
iii. adding a size smaller than the largest serving size to existing options

Thus far, only the first of these has been studied in relation to alcoholic drinks. In the first study to be conducted in real-world settings, removing the largest serving size of wine by the glass (most often 250ml) for four weeks decreased wine sales – a proxy for consumption – across 21 licenced premises by 7.6% [39]. This reflects findings from two prior studies in semi-naturalistic settings, in which the largest servings of wine and beer were removed and replaced with smaller servings. In both studies, the intervention reduced the volume of alcohol consumed on a single occasion [40].

Although potentially effective, interventions that involve restricting options, such as removing the largest serving sizes, are likely to evoke opposition both from the alcohol industry, given their potential to reduce sales of targeted drinks [41], and from the public, who tend to support such interventions less than information-based interventions [42]. Such opposition is arguably less likely with interventions that involve increasing existing options by adding new smaller serving sizes, with larger sizes remaining available. Such interventions do not restrict options and are therefore more likely to be perceived as acceptable to the public [42]. A small number of studies have evaluated these types of interventions in the context of food consumption. Three of these [43–45] found that adding smaller servings of hot meals and entrees to the menus of worksite cafeterias and restaurants resulted in a small but potentially meaningful proportion of customers choosing the smaller servings (between 5%–13% in worksite cafeterias and 19%–31% in restaurants). Although none of these studies assessed the amounts of food or energy purchased, one of them showed – by measurement of leftovers – that the intervention reduced overall energy consumed [45]. Finally, a more recent study found that adding a medium and a smaller serving size of packaged sausages to the default larger size offered in a supermarket reduced the amount of meat purchased [46]. No studies, however, have assessed the impact on alcohol consumption or sales of adding smaller serving sizes of drinks to existing options. In theory, the addition of a smaller serving size to a range of options could reduce alcohol consumption by better reflecting people's existing preferences for an ideal serving size [47, 48]. This would be the case when the largest serving sizes are considered too large but the smaller ones too small. It could also shape norms about what is an appropriate size [49].

The aim of the current study was to assess the impact on beer and cider sales of adding a serving size of draught beer and cider (2/3 pint) to the range of options available in licensed premises that was between the smallest (1/2 pint) and largest (1 pint) serving sizes. We hypothesised that adding a 2/3 pint serving size would reduce the volume of beer and cider sold.

## Methods

The study protocol and statistical analysis plan were pre-registered (ISRCTN: ISRCTN33169631 https://doi.org/10.1186/ISRCTN33169631 (registration date 08/09/2021) Open Science Framework: registration https://osf.io/xkgdb/ (registration date 08/09/2021) protocol: https://osf.io/sxe9t; statistical analysis plan: https://osf.io/9sr5j).

### Study design

The study used an ABA treatment reversal design consisting of three consecutive four-week periods, in which A represented the non-intervention periods during which the usual range of serving sizes was available, and B represented the intervention period.

### Setting and context

The study was conducted in pubs, bars and restaurants in England.

### Participants

Participants were 22 licensed premises in England. Their location and other characteristics are shown in Table 1. Approximately half (54%) were pubs, and were located in London (59%).

**Table 1:** Characteristics of recruited licensed premises

Premises number	Location	Index of Multiple Deprivation Quintile^a^	Premises type^b^	Baseline daily revenue (£) (mean (sd))	Recruitment date
1	Lewisham, London	1	Pub	1977.5 (1265.6)	September 2021
2	Camden, London	3	Pub	1142.3 (821.8)	September 2021
3	Cambridge	5	Bar & Restaurant	4545.5 (2764.9)	September 2021
4	Newham, London	3	Bar	1059.5 (1056.7)	February 2022
5	Birmingham	2	Restaurant	3223.8 (2367.3)	February 2022
6	Camden, London	3	Bar	2967.3 (2110.8)	February 2022
7	Bristol	1	Pub	2206.0 (1228.7)	February 2022
8	Ealing, London	5	Pub	341.0 (159.9)	February 2022
9	Sheffield	2	Bar & Restaurant	4599.7 (4327.1)	February 2022
10	Sheffield	2	Bar	994.5 (818.5)	February 2022
11	Bristol	4	Pub	1820.3 (792.6)	February 2022
12	Wandsworth, London	2	Bar & Restaurant	5540.0 (2790.3)	February 2022
13	Hillingdon, London	3	Pub	1853.1 (1089.7)	February 2022
14	Westminster, London	4	Pub	N/A^c^	February 2022
15	Sheffield	2	Pub	N/A^c^	February 2022
16	Richmond upon Thames, London	3	Bar & Restaurant	N/A^c^	February 2022
17	Sheffield	3	Bar	N/A^c^	February 2022
18	Sheffield	2	Bar	N/A^c^	February 2022
19	Islington, London	2	Pub	N/A^c^	September 2021
20	Greenwich, London	2	Pub	N/A^c^	September 2021
21	Bromley, London	4	Pub	N/A^c^	September 2021
22	Bromley, London	4	Pub	N/A^c^	September 2021

^a^ The Index of Multiple Deprivation (IMD) ranks every area in England according to deprivation levels. The IMD combines information from seven domains to produce an overall relative measure of deprivation; 1 = most deprived; 5 = least deprived

^b^ Description of premises type taken from each premises’ website

^c^ Premises discontinued participation before providing data

To be eligible to take part in the study, licensed premises had to meet the following criteria:

i. sell a minimum of 100 pints of beer and cider on average per week
ii. be willing to introduce 2/3 pints for all beers and ciders sold on tap
iii. have an electronic point of sale (EPOS) till system to record daily sales of all drinks and their served sizes
iv. be primarily indoor, permanent establishments in a fixed location; *i.e.* not purposefully temporary or time-limited (*e.g.* pop-up), or mobile venues (*e.g.* vans)

#### Sample size calculation

Power simulations [50] suggested that 14 sites using an ABA reversal design with each period lasting four weeks would be needed in order to have 80% power to detect a predicted effect of 8.4% reduction in log beer and cider volume. Recruitment was conducted in two waves. Due to a large level of attrition during the first wave, we overrecruited in the second wave. The final recruited sample consisted of 22 sites.

### Intervention

Licensed premises added a new serving size of 2/3 pints to their existing range of draught beers and ciders. The 2/3 pint was offered in addition to the existing larger serving size of one pint and smaller serving size of half-pint, with proportionate pricing, *i.e.* with a price which was linear-by-volume between the pint and half-pint sizes, as confirmed during fidelity checks. The researchers provided the 2/3 pint glasses. Premises adopted a range of strategies to promote the new serving size, including signs, posters, advertising on blackboards and adding it to menus. These were not controlled by the researcher team.

Within the TIPPME intervention typology for changing environments to change behaviour [51], the type of intervention used in the current study was ‘Size’, and focused on the ‘Product’ itself, *i.e.* the alcoholic drink. The TIPPME intervention typology provides a framework to reliably classify and describe interventions which alter small-scale (proximal) physical micro-environments to change selection, purchase and consumption of food, alcohol and tobacco products. In the typology, interventions are described according to two dimensions: type of intervention (changing the Availability, Position, Functionality, Presentation, Size, Information) as well as the spatial focus of the intervention (whether a Product, a Related Object or the Wider Environment is targeted) [51].

### Procedure

Data were collected during two phases: between September 2021 and December 2021 and between February 2022 and May 2022. There were no COVID restrictions in place during the study. Potentially eligible premises were identified through a publicly available database (http://whatpub.com). Those based in one of eight geographical locations where it was feasible for the research team to conduct fidelity checks were sent email invitations to participate in the study. Premises replying with an interest in taking part were sent additional information about the study and were assessed for eligibility over the telephone. Eligible premises agreeing to participate provided written informed consent for doing so.

Recruited premises changed their available serving sizes for draught beer and cider on two occasions over a period of 12 weeks, first to add a 2/3 pint serving size during the intervention period (B), and second to remove it during the second non-intervention period (A). Till systems, menus and signs were updated as appropriate to reflect the available serving sizes. Premises managers were reminded via email one day before each required change.

Premises were paid £250 (plus 20% VAT) for participating in the study and providing all requested data. They were also allowed to keep the 2/3 pint glasses and were reimbursed for the costs of any necessary changes to menus and signs.

### Measures

#### Primary outcome

Daily volume (in milliliters (ml)) of all beer and cider sold (draught as well as bottled), extracted from sale records.

#### Secondary outcomes

The following outcomes were extracted from sales records:

i Daily volume (in ml) of beer and cider sold by each serving size:
  1/3 pint (189ml)
  1/2 pint (284ml)
  330ml bottle
  440ml can
  500ml bottle
  pint (568ml)
  600 ml
  5 L (5000 ml)
ii Daily volume (in ml) of wine sold
iii Daily revenue from food, alcoholic and non-alcoholic drinks

#### Covariates

Given that daily temperature, day of the week, season and holidays can influence alcohol sales [52, 53], the following covariates were considered:

i. Maximum daily local temperature
ii. Dummy variables for special events (e.g. Bank Holidays, other holidays, major sporting events, etc.)
iii. Total revenue (as a proxy for premises busyness)
iv. Dummy variables for day of the week
v. Study day from start of a period (number from 1 to 84)
vi. Season at start of study: autumn or winter

### Data analysis

Unadjusted summaries of the volume of beer and cider sold during the non-intervention and intervention periods were calculated both overall and for each serving size. Outliers in the daily data were identified using range checks, scatter plots, median absolute deviation values and histograms. The potential outliers identified were all deemed genuine values and it was assumed that the two model covariates (total revenue – a proxy for site busyness – and special events) could handle these to ensure no outliers in the model residual diagnostics.

#### Primary analysis

A generalized linear mixed model (being generalized additive models which can accommodate heterogeneity) was used to predict daily volume of beer and cider sales according to study period (A vs B). Premises were treated as a random factor and heterogeneity between premises was modelled. The analysis included pre-specified covariates for day of the week, study day and total revenue. An overall effect was estimated from this model. The mean difference and associated 95% confidence intervals (CI) and p-value, as well as a Cohen’s d effect size and its 95% CI were calculated. All regression model diagnostics (residual plots, worm plots) were checked and were satisfactory once a variance stabilising transformation was used (square root).

Only premises that completed the study per protocol and provided all primary outcome data were included in the primary analysis.

#### Sensitivity analyses

Four sets of sensitivity analyses were conducted to check the robustness of the primary analysis:

1. Generalized linear regression analysis, repeating the primary analysis but taking into account three additional covariates: *i)* total number of special events in each period; ii) season at the start of the study; iii) maximum daily local temperature.
2. Generalized linear regression analysis, repeating the primary analysis but adding daily-level data from all premises, including those that violated the protocol for intervention implementation (intention to treat analysis).
3. Generalized linear regression analysis, repeating the primary analysis but including the two non-intervention periods as separate factor levels (i.e. using A1, B & A2 levels for the periods). This was conducted to assess whether there were differences in the two A periods and whether aggregating the data from these two periods in the primary analysis was justified.
4. As data might be less variable when aggregated at the period level, a generalized linear regression analysis using period-level data to compare mean daily sales during period A (aggregate value for 2 four-week A period) and mean daily sales during period B (aggregate value for 1 four-week B period).

#### Secondary analyses

For the secondary outcomes, generalized linear mixed models were used, with the distribution of the data assessed by model diagnostics dictating which model was most appropriate (*e.g.* Poisson regression).

The following secondary analyses were conducted:

1. Regression analyses to predict the number of beer and cider drinks sold in each serving size according to the study Period (A vs B).
2. A regression analysis to predict the daily volume of wine sold according to the study Period (A vs B). The analysis included covariates for day of the week, study day and total revenue.
3. A regression analysis to predict total revenue from all food and drink according to the study Period (A vs B). The analysis included covariates for day of the week and study day.

## Results

**Fig. 1:**
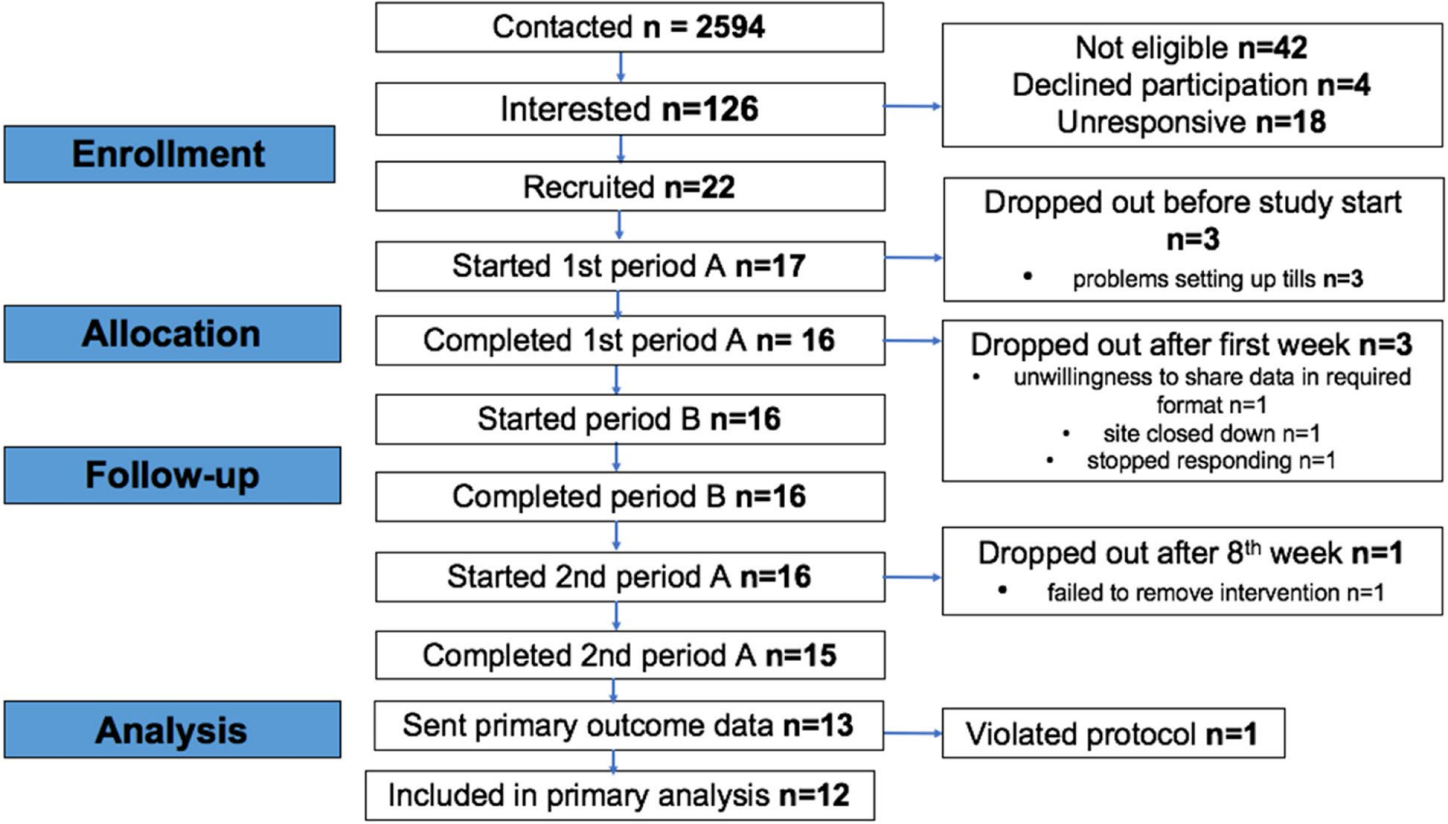
Flow of premises through study

The flow of premises through the study is shown in Fig. 1. Twenty-two licensed premises were recruited from 2594 that were contacted, a recruitment rate of 0.8%. Thirteen completed the study and provided all primary outcome data, a retention rate of 59%. One premises was excluded from the primary analysis for violating the protocol and continuing to sell 2/3 pints during the second non-intervention period, as identified by inspection of their sales data.

### Primary outcome: volume of beer and cider sales

**Fig. 2:**
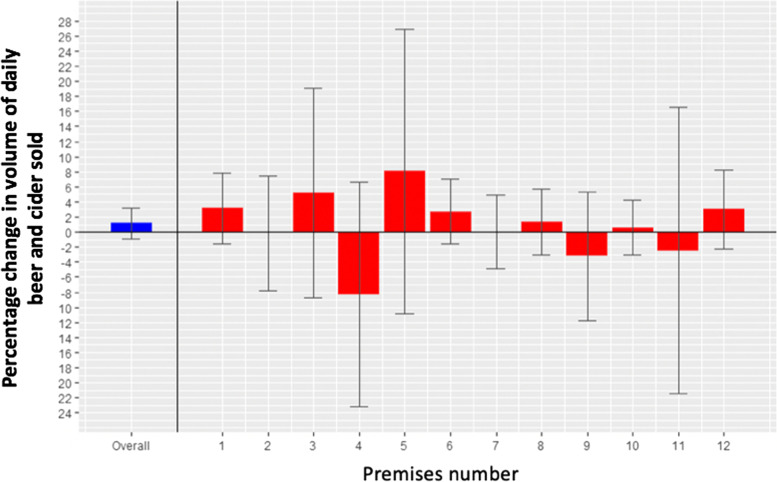
Percentage change in sales of beer and cider from adding a smaller (2/3 pint) serving size

The unadjusted mean daily volume of beer and cider sold per premises during the non-intervention periods (A) was 83,179.32ml (sd = 92,549.14) and 81,296.28ml (sd = 94,259.47) during the intervention period (B). After accounting for pre-specified covariates, the difference in the volume of beer and cider sold per day during the intervention period (B) compared to the volume sold during the two non-intervention periods (A) was not significantly different (square root of volume = 3.14ml, 95%CIs -2.29 to 8.58; *p* = 0.257) (Table 2). There was heterogeneity between premises (sigma coefficients were statistically significant at *p* < 5%; Supplement, Table S1). Figure 2 shows the effect of the intervention on beer and cider sales overall and for each premises (Supplement, Table S1).

**Table 2:** Mixed effects regression results (95% CI) predicting the square root of volume (ml) of beer and cider per day (*n* = 12)

				**95%CI for estimate**	
	**Estimate (SE)**	**t-value**	***P*-value**	**Lower**	**Upper**
Intercept	134.02 (4.24)	31.59	< 0.001	125.70	142.33
Study period (ref: baseline)	3.14 (2.77)	1.13	0.257	-2.29	8.58
Day of the week_Tuesday (ref: Monday)	7.68 (4.32)	1.78	0.0760	-0.79	16.17
Day of the week_Wednesday (ref: Monday)	25.28 (4.51)	5.61	< 0.001**	16.44	34.12
Day of the week_Thursday (ref: Monday)	35.79 (4.92)	7.26	< 0.001**	26.13	45.45
Day of the week_Friday (ref: Monday)	73.06 (5.36)	13.63	< 0.001**	62.56	83.57
Day of the week_Saturday (ref: Monday)	51.55 (7.87)	6.55	< 0.001**	36.12	66.98
Day of the week_Sunday (ref: Monday)	11.65 (5.07)	2.29	0.0219*	1.71	21.59
Study day	0.26 (0.06)	4.56	< 0.001**	0.15	0.37
Total revenue	0.03 (0.00)	37.11	< 0.001**	0.04	0.04

^*^Significant at the *P* < 0.05 level; **significant at the *P* < 0.01 level. *CI* Confidence interval, *SE* Standard error

### Sensitivity analyses

Results were unchanged when performing an intention‐to‐treat analysis (*n* = 13) that included the one premises that had violated the protocol (Table S2, supplement), and when including additional covariates in the model (Table S3). Using period-level data rather than daily-level data, also did not show a significant difference in the volume of beer and cider sold during the intervention compared to the non-intervention periods (-265.3ml; 95% CIS -6756.68 to 6226.01; *p* = 0.93).

Daily sales of beer and cider did not significantly differ during the two non-intervention periods (A) (Table S4, supplement), justifying the modelling choice of combining data from both non-intervention periods for the primary analysis.

### Secondary outcomes

#### Beer and cider sales by serving size

The unadjusted mean daily volume of beer and cider sold in different serving sizes is show in Table 3.

**Table 3:** Unadjusted mean (sd) volume of beer and cider (ml) sold per day, overall and according to serving size (*n* = 13) and results of regression analyses assessing differences in the number of beers and ciders sold in each size according to study period

	**Non-intervention **(both combined)	**Intervention**	**Regression analyses results**
Overall volume of beer/cider sold (ml)	83,179.32 (92,549.14)	81,296.28 (94,259.47)	
Volume of beer/cider sold (ml) in 1/3pints (189ml)	16.39 (106.89)	18.21 (149.32)	0.611 95% CIs 0.149 to 1.07; *p* < 0.01
Volume of beer/cider sold (ml) in ½ pints (284ml)	3402.93 (3716.05)	3127.90 (3729.24)	-0.018 95%CIs -0.088 to 0.053; *p* = 0.62
Volume of beer/cider sold (ml) in 330ml	2021.25 (4181.84)	1746.09 (3239.24)	-0.010; 95% CIs -0.150 to 0.130; *p* = 0.887
Volume of beer/cider sold (ml) in 2/3pints (378ml)	0 (0)	1248.46 (2066.01)	
Volume of beer/cider sold (ml) in 440ml	226.04 (734.49)	228.46 (765.54)	0.042 95%CIs -0.136 to 0,220; *p* = 0.64
Volume of beer/cider sold (ml) in 500ml	1297.39 (3212.31)	1239.01 (1837.93)	0.121 95% CIs -0.0003 to 0.2442; *p* = 0.051
Volume of beer/cider sold (ml) in pints (568ml)	68,605.50 (85,338.76)	66,334.29 (86,441.33)	0.092 95% CIs -0.118 to 0.302; *p* = 0.39
Volume of beer/cider sold (ml) in 600ml	6611.54 (28,756.20)	6932.31 (29,478.25)	-0.087 95% CIs -0.549 to 0.373; *p* = 0.708
Volume of beer/cider sold (ml) in 5L	982.14 (4694.63)	961.54 (4488.57)	0.004 95% CIs -0.116 to 0.124; *p* = 0.949

A Poisson regression (for skewed data) showed that the number of 1/3 pints sold increased during the intervention compared to the non-intervention periods (IRR = with 1.84; 0.611 95% CIs 0.149 to 1.07; *p* < 0.01). However, very few 1/3 pints were sold overall during the study. Additional analysis revealed no evidence that the proportion of days during which any 1/3pints were sold varied according to study period (-0.22; 95%CIs -1.16 to 0.64; *p* = 0.62).

Negative binomial regressions (for skewed data) found no evidence that the number of beers and ciders sold in the following serving sizes differed according to study period: 1/2 pints (-0.018 95%CIs -0.088 to 0.053; *p* = 0.62); 440ml (0.042 95%CIs -0.136 to 0,220; *p* = 0.64); 600ml (-0.087 95% CIs -0.549 to 0.373; *p* = 0.708).

Mixed effect regressions found no evidence that the number of beers and ciders sold in the following serving sizes differed according to study period: 330ml (-0.010; 95% CIs -0.150 to 0.130; *p* = 0.887); 500ml (0.121 95% CIs -0.0003 to 0.2442; *p* = 0.051); pint (0.092 95% CIs -0.118 to 0.302; *p* = 0.39); 5L (0.004 95% CIs -0.116 to 0.124; *p* = 0.949).

A regression estimating the daily volume of 2/3 pints sold during the intervention period only showed that this was significantly different from zero (square root of volume = 1.30 95% CI 1.22 to 1.38 *p* < 0.001).

#### Volume of wine sold

The unadjusted mean daily volume of wine sold during the non-intervention periods (A) was 4273.2ml (sd = 5291.9) and 3979.6ml (sd = 4579.9) during the intervention period (B). There was no evidence of a difference in the volume of wine sold per day between the intervention and non-intervention periods (square root of volume = -1.00, 95%CIs -3.48 to 1.46; *p* = 0.424).

#### Revenue

The unadjusted mean daily revenue during the non-intervention periods (A) was £2487.6 (sd = £2504.2) and £2336.0 (sd = £2370.6) during the intervention period (B). There was no evidence of a difference in total daily revenue (£) between the intervention and non-intervention periods (square root of revenue = -0.133, 95% CI -1.126 to 0.860; *p* = 0.793).

## Discussion

There was no evidence in licensed premises that adding a smaller serving size (2/3 pint) that was between the smallest and largest sizes to the existing options for draught beer and cider affected the volume of beer and cider sold. There was also no evidence that wine sales or daily revenues were impacted by the intervention.

These findings did not support the study hypothesis that adding 2/3 pints to existing options of beer and ciders reduces sales. They also appear to differ from findings of the few studies assessing the impact of adding smaller servings on food consumption [43–46]. Three of these studies [43–45] had different outcome measures to the current study –proportion of people selecting smaller servings and/or total energy consumed – which could explain the different conclusions. The one study that used a similar outcome measure to the current study, *i.e.* amount of food purchased, was conducted in a supermarket where customers had physical access to all available serving sizes [46], in contrast to the current study, in which people had to order a serving size in order to see it.

There are two likely explanations for the lack of an evident effect of smaller serving sizes in the current study. First, the current study was underpowered to detect anything other than a large effect of 2/3 pints on beer and cider sales. Studies conducted in real-world settings with greater power to detect smaller but still meaningful effects are therefore warranted.

Second, the findings reflect a true null effect, operating through at least two routes. While some might have shifted from a pint to a 2/3 pint, a similar number might have shifted from a 1/2 pint to a 2/3 pint. Alternatively, the new size was not selected enough to have an effect. Reflecting this, although the volume of beer and cider sold in 2/3 pints during the intervention period differed from zero, it was only 1.5% of the total volume of beer and cider sold. Furthermore, 2/3 pints might not have been selected more often because customers were unaware of the new size, or despite being aware they did not prefer it. Premises adopted a range of strategies to advertise the new serving size, including signs, posters and adding it to menus, which were not evaluated. It is also not known how the new size was promoted by staff. Invitations to downsize serving sizes at the point of sale can shift customers to smaller sizes [54]. Given that premises in the study were likely motivated to sell larger sizes to increase revenue, such strategies might have not been used.

The role of norms is a final factor that may explain the lack of impact of the intervention. People hold social and personal norms for what constitutes an appropriate serving size to consume [49, 55]. The addition of a new serving can shape these social norms but it is possible that in the present study there was not enough time for this to occur. The customary serving size for draught beer and cider in the UK is a pint [56] and has been for centuries [56]. Shifting this well-established norm could require considerably more time than the four weeks the new size was on offer in this study.

### Implications for research and policy

If the intervention assessed in this study is found to have an effect in subsequent, adequately powered studies, changing alcohol licensing regulations to make 2/3 pints for draught beer and cider available mandatorily in licensed premises, in the same way as 125 ml serving sizes of wine are, might be worth consideration. This is especially important given that interventions that involve adding a smaller serving size to existing options are likely to be better accepted by businesses and the public than those that involve removing or restricting options [57–59].

A potentially more effective intervention is to remove the largest serving size from existing options. Removing the largest serving of wine by the glass from sale in licensed premises reduces wine sales by an estimated 6.5%, without affecting total revenues [39]. In the case of draught beer and cider in the UK, the largest and most popular serving size is the pint (568ml) [56] Future research should assess the impact on the volume of beer and cider consumed of removing the offer of pints in licensed premises.

### Strengths and limitations

The strength of the present study is that it is the first to estimate the impact of increasing the available options for alcoholic drinks in licensed premises by adding a smaller serving size, as a method for reducing excess alcohol consumption. A further strength is the use of objective measures of impact. The study, however, has several limitations. First, the generalisability of the findings is limited by the majority of premises studied being in London, and constituting a very small proportion of those approached. Second, the study relied on an A-B-A design, which has a higher risk of bias than experimental designs, although the analyses attempted to account for potential confounding variables. Third, although all premises promoted the introduction of 2/3 pints using posters, advertising on blackboards and making changes to menus, the study neither controlled for nor evaluated the strategies premises adopted. As such, it is not known whether variations in how 2/3pints were advertised could have influenced the findings. Finally, outcomes concerned sales rather than actual consumption, although sales are a valid, practicable, commonly used [60–62] proxy for consumption [60–64].

## Conclusion

In conclusion, increasing the available serving sizes for draught beer and cider in licensed premises by adding a smaller serving size (2/3 pint) when the largest size (1 pint) was still available, did not provide evidence of an effect on the volume of beer and cider sold. Additional studies are warranted with more power to detect smaller effect sizes for the intervention, as well as to assess the impact of removing the largest serving size.

## Supplementary Information

**Additional file 1:** **Additional Methods and Results**

## Data Availability

The dataset generated during the current study is available on the Open Science Framework repository (https://osf.io/evm6u).
