# Supplementary material for: Impact on sales of adding a smaller serving size of beer and cider in licensed premises: an A-B-A reversal design
Source: BMC Public Health. 2023 Jun 26;23:1239. doi: 10.1186/s12889-023-16163-z (PMC10294394; doi:10.1186/s12889-023-16163-z)
Supplement: Supplementary file 1 — Additional file 1: Additional Methods and Results [file 12889_2023_16163_MOESM1_ESM.docx]

**Additional Methods and Results**

1. **Additional Methods**

**Setting and context**

The study was conducted in licensed premises in England, where the sizes of servings of alcoholic drinks sold in licensed premises are subject to regulations^1^. Draught beer and cider must legally be available in one of two sizes^1^: pints (568ml) – which is the most popular measure^2^ – and half pints (284ml). Since 2011, one-third (189ml) and two-third pints (379 ml) can also be sold, but licensed premises are not legally obliged to make these available ^2 3^.

**Sample size calculations**

Power simulations were based on a linear mixed effects model of the daily data of four premises – one restaurant and three student bars – which took part in a pilot study.

Due to a large level of attrition during the first wave (four out of seven recruited sites dropped out: three – owned by the same person- reported problems programming their tills and dropped out before starting the study; one was not able to provide data in the requested format after the first week of the study- we overrecruited in the second wave. Seven of the 22 recruited premises were recruited in the first wave and 15 in the second wave.

**Procedures**

Premises staff were asked not to mention the study to customers. If any customers enquired about the serving size changes, they were given a simple scripted explanation “We have been receiving requests for differently sized drinks, so we are trying out some changes for a few weeks”.

Fidelity to the protocol was checked by a researcher visiting each premises anonymously on two occasions to establish whether the correct serving sizes were on offer first, during the intervention period (B) and second, during the final non-intervention period (A). Premises failing checks were informed within 24 hours and asked to rectify the situation immediately. This was followed by a repeat visit anonymously to establish whether the required change had occurred. Data from the weeks for which checks were failed were disregarded and periods were extended by the equivalent number of disregarded days, to ensure that each period always lasted four weeks and premises completed 12 weeks in the study.

Only premises that met the following three conditions were included in the primary analysis:

1. completed the study in full, *i.e.* all 12 weeks
2. provided primary outcome data for the 12 weeks of the study
3. adhered to the protocol for intervention implementation, *i.e.* they passed the fidelity checks and their data did not suggest that 2/3 pints were sold during Period Β.

**References**

1. Goverment UK. Weights and measures: the law [Available from: <https://www.gov.uk/weights-measures-and-packaging-the-law>.

2. de Moor D. The ultimate beer measures table 2017 [Available from: <https://desdemoor.co.uk/the-ultimate-beer-measures-table/>.

3. BBC. Schooner set to join pint after drinks measures review 2011 [Available from: <https://www.bbc.com/news/uk-12113880>.

**Additional Results**

**Table S1:**

Mixed effects regression results (95% CI) predicting the square root of volume (ml) of beer and cider per day for individual licensed premises (reference Premises 1) (n=12).

|  |  |  |  |
| --- | --- | --- | --- |
|  | **Estimate (SE)** | **t-value** | **P-value** |
| Intercept | 3.212 (0.098) | 32.66 | <0.001 |
| Premises 2 | 0.567 (0.121) | 4.66 | <0.001** |
| Premises 3 | 0.800 (0.120) | 6.66 | <0.001** |
| Premises 4 | 0.408 (0.127) | 3.21 | <0.05* |
| Premises 5 | 0.747 (0.122) | 6.14 | <0.001** |
| Premises 6 | 0.576 (0.112) | 4.92 | <0.001** |
| Premises 7 | -0.207 (0.118) | -1.76 | 0.079 |
| Premises 8 | 0.393 (0.115) | 3.43 | <0.001** |
| Premises 9 | 0.848 (0.113) | 7.52 | <0.001** |
| Premises 10 | 0.368 (0.117) | 3.14 | <0.05* |
| Premises 11 | 1.128 (0.117) | 9.64 | 0.474 |
| Premises 12 | 0.376 (0.114) | 3.29 | <0.05* |
| Day of the week_Tuesday | -0.091(0.092) | -0.99 | 0.319 |
| Day of the week_Wednesday | -0.059 (0.089) | -0.66 | 0.507 |
| Day of the week_Thursday | 0.151 (0.089) | 1.70 | 0.088 |
| Day of the week_Friday | 0.248 (0.089) | 2.75 | <0.05* |
| Day of the week_Saturday | 0.740 (0.093) | 7.92 | <0.001** |
| Day of the week_Sunday | 0.137 (0.093) | 1.48 | 0.139 |

*Significant at the P < 0.05 level; **significant at the P < 0.01 level. CI = confidence interval; SE = standard error.

**Table S2:** Mixed effects regression model estimates of the square root of volume (ml^0.5)

(95% CI) of beer and cider sold per day – intention to treat analysis (n=13).

|  |  |  |  | **95%CI for estimate** | |
| --- | --- | --- | --- | --- | --- |
|  | **Estimate (SE)** | **t-value** | **P-value** | **Lower** | **Upper** |
| Intercept | 134.59 (4.08) | 32.98 | <0.001 | 126.59 | 142.58 |
| Study period (ref: baseline) | 0.71 (2.72) | 0.26 | 0.794 | -4.61 | 6.03 |
| Day of the week_Tuesday (ref: Monday) | 9.10 (4.16) | 2.18 | 0.0760 | 0.94 | 17.26 |
| Day of the week_Wednesday (ref: Monday) | 26.47 (4.34) | 6.10 | <0.001** | 17.97 | 34.97 |
| Day of the week_Thursday (ref: Monday) | 38.03 (4.76) | 7.99 | <0.001** | 28.70 | 47.37 |
| Day of the week_Friday (ref: Monday) | 71.30 (5.41) | 13.18 | <0.001** | 60.70 | 81.90 |
| N Day of the week_Saturday (ref: Monday) | 45.63 (7.96) | 5.73 | <0.001** | 30.02 | 61.23 |
| Day of the week_Sunday (ref: Monday) | 10.64 (4.77) | 2.23 | 0.0260* | 1.29 | 19.99 |
| Study Day | 0.25 (0.05) | 4.61 | <0.001** | 0.14 | 0.36 |
| Total revenue | 0.04 (0.00) | 36.81 | <0.001** | 0.035 | 0.039 |

*Significant at the P < 0.05 level; **significant at the P < 0.01 level. CI = confidence interval; SE = standard error.

**Table S3:** Mixed effects regression model estimates of the square root of volume (ml^0.5)

(95% CI) of beer and cider sold per day – additional covariates (n=12).

|  |  |  |  | **95%CI for estimate** | |
| --- | --- | --- | --- | --- | --- |
|  | **Estimate (SE)** | **t-value** | **P-value** | **Lower** | **Upper** |
| Intercept | 65.18 (7.53) | 8.65 | <0.001 | 50.42 | 79.93 |
| Study period (ref: baseline) | 2.61 (2.81) | 0.93 | 0.352 | -2.89 | 8.11 |
| Day of the week_Tuesday (ref: Monday) | 7.06 (4.35) | 1.62 | 0.104 | -1.46 | 15.59 |
| Day of the week_Wednesday (ref: Monday) | 25.66 (4.52) | 5.68 | <0.001** | 16.81 | 34.51 |
| Day of the week_Thursday (ref: Monday) | 36.45 (4.93) | 7.39 | <0.001** | 26.78 | 46.12 |
| Day of the week_Friday (ref: Monday) | 74.07 (5.46) | 13.58 | <0.001** | 63.38 | 84.77 |
| N Day of the week_Saturday (ref: Monday) | 51.73 (8.07) | 6.41 | <0.001** | 35.92 | 67.55 |
| Day of the week_Sunday (ref: Monday) | 14.24 (5.23) | 2.72 | 0.006** | 3.99 | 24.49 |
| Study Day | 0.25 (0.06) | 4.43 | <0.001** | 0.14 | 0.37 |
| Total revenue | 0.04 (0.00) | 36.54 | <0.001** | 0.03 | 0.04 |
| Special event | 2.72 (7.73) | 0.35 | 0.7246 | -12.42 | 17.87 |
| Max daily temperature | 1.66 (0.37) | 4.45 | <0.001** | 0.93 | 2.39 |
| Start of study season (ref: winter) | 61.17 (3.20) | 19.09 | <0.001** | 54.89 | 67.45 |

*Significant at the P < 0.05 level; **significant at the P < 0.01 level. CI = confidence interval; SE = standard error.

**Table S4:** Mixed effects regression model estimates (95% CI) of the square root of volume (ml^0.5) of beer and cider sold per day (n=12) – separating non-intervention periods

|  |  |  |  |
| --- | --- | --- | --- |
|  | **Estimate (SE)** | **t-value** | **P-value** |
| Intercept | 134.0 (4.4463) | 30.138 | <0.001 |
| Intervention (ref: first non-intervention) | 3.0751 (5.8136) | 0.529 | 0.05970 |
| Second non-intervention (ref: firs non-intervention) | -0.138183 (10.2436) | -0.013 | 0.9892 |
| Day of the week_Tuesday (ref: Monday) | 7.688285 (4.329838) | 1.776 | 0.0761 |
| Day of the week_Wednesday (ref: Monday) | 25.283363 (4.512257) | 5.603 | <0.001** |
| Day of the week_Thursday (ref: Monday) | 35.782987 (4.962173) | 7.211 | <0.001** |
| Day of the week_Friday (ref: Monday) | 73.058477 (5.404027) | 13.519 | <0.001** |
| N Day of the week_Saturday (ref: Monday) | 51.547126 (7.895840) | 6.528 | <0.001** |
| Day of the week_Sunday (ref: Monday) | 11.638281 (5.152636) | 2.259 | <0.05** |
| Study Day | 0.261413 (0.170145) | 1.536 | 0.1248 |
| Total revenue | 0.039143 (0.001056) | 37.0.61 | <0.001** |

*Significant at the P < 0.05 level; **significant at the P < 0.01 level.

For the sensitivity analysis that used period-level data, mean daily sales for each period were calculated by adding the total volume of beer and cider sold and dividing by the number of days the premises were open during each A and B period.
